# Mitochondria-target ubiquinone attenuates bleomycin-induced pulmonary fibrosis

**DOI:** 10.3389/fphar.2025.1661644

**Published:** 2025-09-04

**Authors:** Ying Jiang, Zhenghui Huang, Ting Zhou, Mi Wu, Juan Zhao, Zheyi Xiong, Rui Wang, Limin Chen, Xiufang Weng, Lan Lin

**Affiliations:** ^1^ Department of Immunology, School of Basic Medicine, Tongji Medical College, Huazhong University of Science and Technology, Wuhan, China; ^2^ Department of Pulmonary and Critical Care Medicine, Fujian Medical University Union Hospital, Fuzhou, Fujian, China; ^3^ Key Laboratory of Organ Transplantation, Ministry of Education, NHC Key Laboratory of Organ Transplantation, Key Laboratory of Organ Transplantation, Chinese Academy of Medical Sciences, Wuhan, China

**Keywords:** pulmonary fibrosis, bleomycin, oxidative stress, pro-fibrotic molecule, mitochondria-targeted ubiquinone (MitoQ)

## Abstract

**Background:**

Pulmonary fibrosis arises from various etiologies, often associated with elevated levels of reactive oxygen species (ROS) stress and activation of pro-fibrotic signaling pathways. The chemotherapeutic drug bleomycin has been shown to exacerbate pulmonary fibrosis during anti-tumor treatment. Further research is needed to combat bleomycin-induced fibrosis.

**Aim:**

This investigation aims to identify critical mediators of bleomycin-induced pulmonary fibrosis and evaluate the therapeutic potential of mitochondria-targeted ubiquinone (MitoQ) in attenuating fibrotic pathogenesis.

**Methods:**

A bleomycin-induced pulmonary injury mouse model and fibroblast cell culture were established, followed by histopathology evaluation, molecule interaction analysis, cytokine quantification, intervention assay, and flow cytometry.

**Results:**

We analyzed RNA-seq data from a bleomycin-induced pulmonary fibrosis mouse model and identified a network of oxidative stress-related fibrosis genes centered on Tgfb1. In fibroblast cell lines, bleomycin exposure elevated mitochondrial and cellular ROS, increased mitochondrial mass and the MDR^low^/MTG^high^ cell ratio, downregulated genes linked to ROS scavenging and mitochondrial function, and upregulated transcription of pro-fibrotic molecules. MitoQ effectively reduced mitochondrial ROS, alleviated mitochondrial swelling, and restored transcription of genes involved in mitochondrial redox balance and function. Compared to conventional ubiquinone, MitoQ exhibited significantly greater antifibrotic efficacy, effectively attenuating bleomycin- and TGF-β1-induced fibroblast activation *in vitro*. In bleomycin-treated mice, MitoQ treatment with markedly suppressed pro-fibrotic molecule transcription and inhibited pulmonary fibrosis progression.

**Conclusion:**

These findings not only advance our understanding of the interplay between oxidative stress and pro-fibrotic signaling in bleomycin-induced pulmonary fibrosis but also provide experimental data supporting the use of mitochondria-targeted antioxidant in the treatment of this condition.

## Introduction

The incidence of pulmonary fibrosis is increasing every year, placing a significant financial burden on healthcare system and emerging as a global public health concern (Collard et al., 2015; Hutchinson et al., 2015). In particular, the increased incidence of diffuse pulmonary fibrosis in patients receiving various forms of cancer chemotherapy has implicated the lung as primary target for a number of cytotoxic agents. Bleomycin, an important chemotherapeutic glycopeptide antibiotic initially isolated from *Streptomyces verticillatus* in Japan, has been widely used in treating several types of tumors, including lymphoma, testicular carcinoma, non-seminomatous germ cell tumors (NSGCTs), and ovarian cancer (Constantin and Widmann, 2020; Harstrick et al., 1991; Vidal et al., 2015; Hiester et al., 2021). Similar to other non-specific chemotherapy agents, bleomycin is associated with significant side effects, particularly pulmonary toxicity, which can be fatal. Pulmonary complications arise in up to 40% of patients receiving therapies containing bleomycin, resulting in mortality rates ranging from 1% to 4% (Constantin and Widmann, 2020). Bleomycin-induced pulmonary fibrosis is a progressive and irreversible disease intricately linked to oxidative stress, mitochondrial DNA damage and cell death (Chen and Stubbe, 2005).

Recent studies have elucidated the role of reactive oxygen species (ROS) in specific fibrotic processes, including macrophage polarization, alveolar epithelial cell apoptosis, myofibroblast differentiation, and changes in the acellular extracellular matrix (Pei et al., 2022; Otoupalova et al., 2020). Given that oxidative stress influences multi-facets of the pathophysiology of pulmonary fibrosis, it has emerged as a promising therapeutic target for exploring combination therapeutic interventions (Otoupalova et al., 2020; Ornatowski et al., 2020). Evidence suggests that antioxidants targeting ROS may attenuate fibrosis in various organs (Qiu et al., 2019; Pan et al., 2021; Diebold and Jain, 2023). N-acetylcysteine (NAC), a known antioxidant, has been reported to enhance the immune response and inhibit epithelial-mesenchymal transition to alleviate pulmonary fibrosis in chronic obstructive pulmonary disease (Zhu et al., 2021). However, one clinical trial indicated that the NAC group did not alleviate idiopathic pulmonary fibrosis compared with the placebo group (Raghu et al., 2012).

Cellular reactive oxygen species (ROS) are primarily generated in mitochondria during oxidative phosphorylation (OXPHOS) within the electron transport chain. Coenzyme Q (CoQ, or ubiquinone) plays a critical role in this process. As a lipid-soluble electron carrier in the inner mitochondrial membrane, CoQ shuttles electrons between complex I/II and complex III of the electron transport chain, facilitating ATP production. During OXPHOS, partial reduction of CoQ can lead to superoxide anion formation—one of the main sources of mitochondrial ROS. Conversely, CoQ also acts as an antioxidant, scavenging ROS and protecting mitochondrial lipids and proteins from oxidative damage, highlighting its dual role in both energy metabolism and redox balance. Mitochondria-targeted ubiquinone (MitoQ) consists of a lipophilic cation (triphenylphosphonium, TPP+) attached to ubiquinone. The TPP moiety allows MitoQ to across the mitochondrial membrane and concentrate in the mitochondrial matrix due to the membrane’s negative potential (Smith and Murphy, 2010; Goh et al., 2019). MitoQ’s unique composition allows it to specifically target mitochondria where it can efficiently exert its antioxidant effects. Its ability to accumulate in high concentrations in mitochondria further enhances its effectiveness in combating oxidative damage (Gioscia-Ryan et al., 2018). Extensive research has highlighted MitoQ’s anti-inflammatory and antioxidant properties in various diseases, including cardiac fibrosis and non-Alcoholic Steatohepatitis (Goh et al., 2019; Turkseven et al., 2023). Overall, MitoQ shows promise as a therapeutic agent for the treatment of conditions characterized by oxidative stress. In a previous study conducted in our laboratory, we demonstrated that the MitoQ reversed concanavalin A-induced liver dysfunction and fibrosis (Desta et al., 2020). Whether MitoQ exerts a similar protective effect in bleomycin-induced pulmonary fibrosis remains uncertain.

In the current study, the findings reveal increased ROS stress associated with Tgfb1-centered pro-fibrotic network in the bleomycin-induced lung injury and fibrosis mouse model. MitoQ, surpassing the effects of standard ubiquinone, protects from bleomycin-induced pulmonary fibrosis and reduces TGF-β1-induced oxidative stress-associated pro-fibrotic molecules upregulation.

## Materials and methods

### Animal studies

C57BL/6J mice (6–8 weeks old, 22–25 g) were purchased from Vital River Laboratories (Beijing, China). All animal experiments were conducted in compliance with the regulations of the Institutional Animal Care and Use Committee and were approved by the Laboratory Animal Welfare & Ethics Committee of Fujian Medical University (Approval No: IACUC FJMU 2024–0065). The mice were randomly assigned four groups and anesthetized with pentobarbital sodium (50 mg/kg, intraperitoneal injection). The groups received different treatments via intraperitoneal injections very other day: saline (Vehicle), Mitochondria-targeted ubiquinone (2.5 mg/kg, CAS No: 845959–50-4, MCE, China) (MitoQ), bleomycin (2.5 U/kg, CAS No: 9041–93-4, MCE, China) (BLM, bleomycin), or bleomycin + MitoQ (BLM + MitoQ). Body weight was monitored every 2 days. After 21 days, e mice were euthanized, their lungs were harvested and weighed, and the lung organ coefficient was calculated using the formula: lung organ coefficient = lung tissue mass (g)/animal body weight (g) × 100%, as previously described (Xiong et al., 2021).

### HE staining, and masson’s trichrome staining in pulmonary tissue

The left lung tissues of the mice were collected, fixed in 4% paraformaldehyde, embedded in paraffin, and sectioned. The sections were stained with hematoxylin–eosin or subjected to Masson’s trichrome staining according to the manufacturer’s instructions (Servicebio Technology, China). Morphologic and pathologic changes in lung sections were evaluated and scored using the Ashcroft score system, as previously described (Hübner et al., 2008). Semi-quantitative analysis was performed using ImageJ software, and the collagen volume fraction (CVF) for each sample was calculated as the collagen area of the field divided by the total area of the field, as previously described (Geng et al., 2022).

### Functional annotation and pathway enrichment analysis

The RNA sequencing data of lung tissue from bleomycin-induced fibrosis (n = 3) versus saline controls (n = 3) were sourced from the GSE25640 dataset and submitted to a secondary analysis to identify differentially expressed genes (DEGs) using GEO2R (http://www.ncbi.nlm.nih.gov/geo/geo2r). DEGs were identified based on criteria of adjusted p-values ≤0.05 and |fold change| ≥ 1, followed by Gene Set Enrichment Analysis (GSEA) and visual mapping via the online easyGSEA tool (https://tau.cmmt.ubc.ca/eVITTA/easyGSEA/).

### Protein-protein interaction prediction analysis

The lists of oxidative stress genes and lung fibrosis genes were separately downloaded from the GeneCards database (https://www.genecards.org) and the Pulmonary Fibrosis gene set of Comparative Toxicogenomics Database (https://ctdbase.org/) as previously reported (Chang et al., 2023; Wang et al., 2022; Zhang et al., 2023; Ruf et al., 2023). The common DEGs among oxidative stress-related genes and lung fibrosis-related genes were imported into STRING (https://cn.string-db.org/) and Cytoscape to construct a protein-protein interaction network, followed by visual mapping for interaction analysis and identification of key regulatory nodes.

### Quantitative real-time reverse transcription-polymerase chain reaction (qRT-PCR)

RNA was extracted from tissues using Trizol Isolation Reagent (15596026CN, Invitrogen, Thermo Fisher Scientific, USA) according to the manufacturer’s protocol. The extracted RNA was reverse transcribed into cDNA using the PrimeScript™ RT Reagent Kit with gDNA Eraser (Code No. RR047A; Takara, Takara Biomedical Technology, China). The cDNA products were then subjected to qRT-PCR analysis using the primer sets listed in Table 1, along with TB Green^®^ Premix Ex Taq™ II (Code No. RR820A; Takara, Takara Biomedical Technology, China) according to the manufacturer’s protocol. Ct values of the target genes were normalized to the Ct values of Gapdh in mouse samples and fibroblast cell (WML2, Chinese Academy of Sciences), and GAPDH in human fibroblast cell line (MRC5, ATCC), and the relative mRNA levels of indicated genes were calculated using the 2^−ΔΔCT^ methods.

**TABLE 1:** Primers for qRT-PCR.

Species	Target gene	Forward 5′- 3′	Reverse 5′- 3′
Ms	α-SMA	GTC​CCA​GAC​ATC​AGG​GAG​TAA	TCG​GAT​ACT​TCA​GCG​TCA​GGA
Ms	COL1a1	GCT​CCT​CTT​AGG​GGC​CAC​T	CCA​CGT​CTC​ACC​ATT​GGG​G
Ms	TGFβ1	CTC​CCG​TGG​CTT​CTA​GTG​C	GCC​TTA​GTT​TGG​ACA​GGA​TCT​G
Ms	Gapdh	CGC​TCC​TGG​AAG​ATG​GTG​AT	GGC​AAA​TTC​AAC​GGC​ACA​GT
MS	Nqo1	TCG​GGC​TAG​TCC​CAG​TTA​GA	AAG​TTA​GTC​CCT​CGG​CCA​TT
Ms	Sod2	CAG​ACC​TGC​CTT​ACG​ACT​ATG​G	CTC​GGT​GGC​GTT​GAG​ATT​GTT
Ms	Cat	CCT​CGT​TCA​GGA​TGT​GGT​TT	AGG​AAT​CCG​CTC​TCT​GTC​AA
Ms	Mfn1	CCT​ACT​GCT​CCT​TCT​AAC​CCA	AGG​GAC​GCC​AAT​CCT​GTG​A
Ms	Mfn2	ACC​CCG​TTA​CCA​CAG​AAG​AAC	AAA​GCC​ACT​TTC​ATG​TGC​CTC
Ms	Drp1	CAG​GAA​TTG​TTA​CGG​TTC​CCT​AA	CCT​GAA​TTA​ACT​TGT​CCC​GTG​A
Homo	ACTA2	AAA​AGA​CAG​CTA​CGT​GGG​TGA	GCC​ATG​TTC​TAT​CGG​GTA​CTT​C
Homo	GAPDH	GGA​GCG​AGA​TCC​CTC​CAA​AAT	GGC​TGT​TGT​CAT​ACT​TCT​CAT​GG

Ms: Mus musculus (Mouse); Homo: Homo sapiens (Human).

### Cytokine quantification

Mouse serum IL-6 levels were determined using Cytometric Bead Array (CBA) Flex Sets (BD Biosciences) according to the manufacturer’s protocol. Data were collected using a FACS Verse cytometer and analyzed using FlowJo software.

### Cell culture

Mouse fibroblast cell line (WML-2) and human fibroblast cell line (MRC-5) were cultured in DMEM medium supplemented with 10% fetal calf serum, 1% penicillin/streptomycin at 37 °C and 5% CO2. Cells (2 × 10^5^) were treated with 10 ng/mL recombinant mouse TGF-β1 (HY-P7117, MCE, China), human TGF-β1 (#100–21, Peprotech, USA) or 1 μg/mL bleomycin (CAS No. 9041–93-4, MCE, China), in the presence of MitoQ (CAS No. 845959–50-4, MCE, China) or coenzyme Q10 (CAS No.C8538, Sigma-Aldrich, USA) for 48 h.

### Detection of reactive oxygen species (ROS) and mitochondrion by flowcytometry

Intracellular and mitochondrial ROS levels were measured using 2,7-dichlorodihydrofluorescein diacetate (DCFH-DA, 5 μM, D6883, Sigma-Aldrich, USA) and MitoSOX Red (5 μM, M36008, Thermo Fisher Scientific, USA), respectively, with incubation at 37 °C for 30 min, as previously reported (Little et al., 2020). Mitochondrial mass and membrane potential were measured using MitoTracker green (MTG, M7514, Invitrogen, USA) and MitoTracker Deep Red (MDR, M22426, Invitrogen, USA) Respectively. Mean fluorescence intensity (MFI) and positively-stained cells were detected using a FACS Verse cytometer (BD Biosciences, USA) and analyzed with FlowJo software.

### Western blot of murine α-SMA, COL1α and TGF-β1

Cell samples were collected, and proteins were extracted using the standard protein extraction protocol. Protein samples were boiled in sodium dodecyl sulfate (SDS)-containing loading buffer for 10 min before loading onto a gel, followed by separation via a SDS-polyacrylamide gel electrophoresis (SDS-PAGE). Proteins were then electrophoretically transferred onto a polyvinylamide fluoride (PVDF) membrane, followed by 5% skimmed milk blocking for 1 h. The membrane was incubated with antibodies against murine α-SMA (A17910, ABclonal, China), COL1α (A22089, ABclonal, China) or TGF-β1 (AB215715, Abcam, UK), and bands were detected and taken photos using a Western blot machine (CLinx Science Instruments).

### Statistical analysis

All statistical analyses were performed using GraphPad Prism software version 8.4.0. Data with normal distribution and equal variance were analyzed by Student’s t-test or ANOVA for group comparisons. For non-normally distributed variables, Mann-Whitney U test or Wilcoxon matched-pairs signed rank test was performed. Correlation analysis was evaluated by Spearman rank correlation. P-values less than 0.05 were considered statistically significant, with * for P < 0.05, ** for P < 0.01, and *** for P < 0.001.

## Results

### Increased oxidant stress and elevated pulmonary fibrosis characteristics in bleomycin-treated mice

**FIGURE 1:**
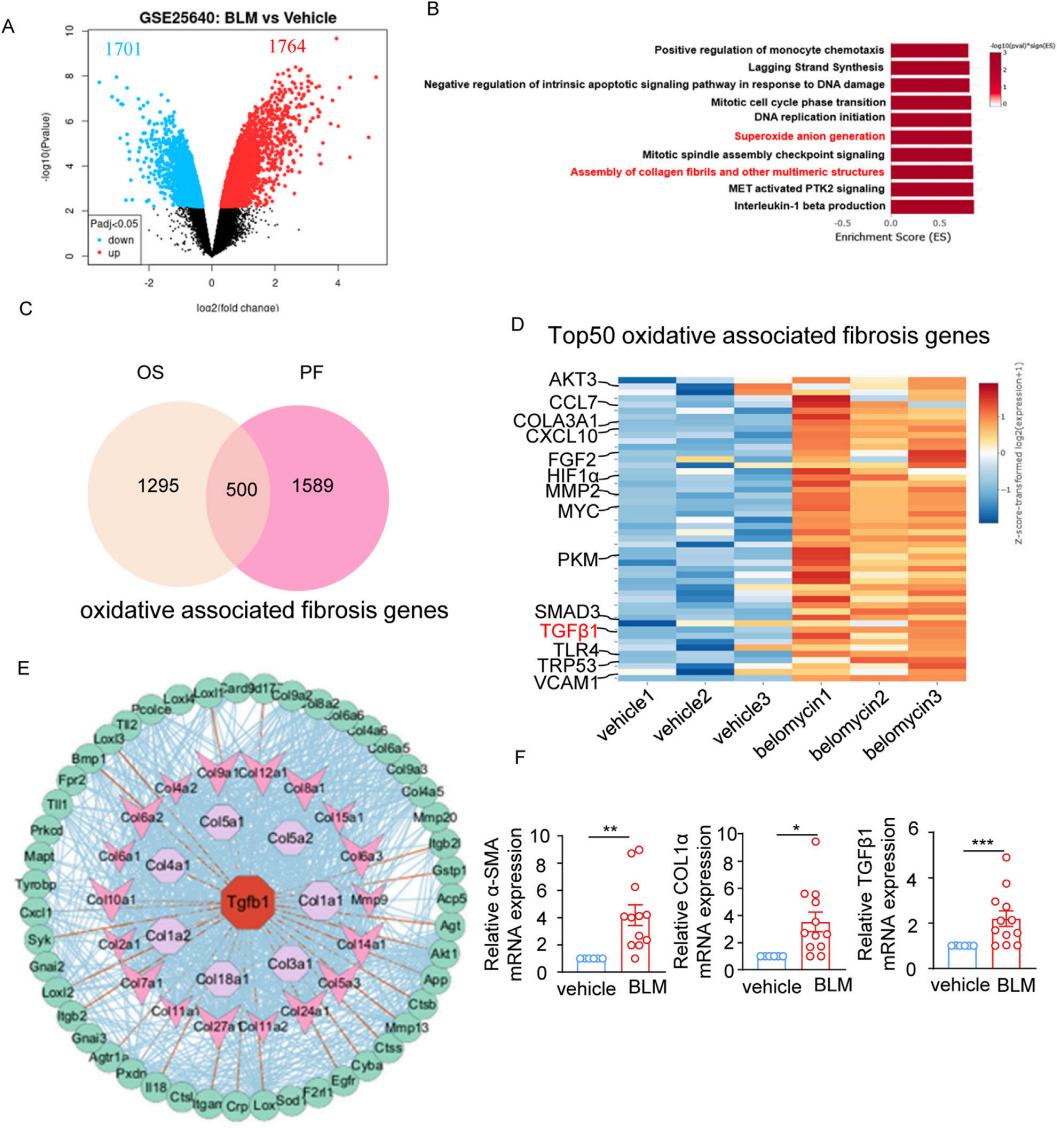
Oxidant stress is associated with pulmonary fibrosis characteristics in bleomycin-treated mice. **(A)** Volcano plot depicting the 1764 upregulated and 1701 downregulated differentially expressed genes (DEGs) in the lung tissue of bleomycin-treated mice (n = 3) compared to controls (n = 3) from the GSE25640 dataset (adjusted p-value cutoff of 0.05). **(B)** The top10 enriched upregulation pathways based on differentially expressed genes. The vertical axis represents the functional annotation information, and the horizontal axis represents the Enrichment Score corresponding to the function (the number of differential genes annotated to the function divided by the number of genes annotated to the function). **(C)** Venn diagram showing 1,295 oxidative stress-related genes and 1,589 pulmonary fibrosis-related genes downloaded from the MitoCarta3.0 database and the Comparative Toxicogenomics Database, and the 500 common genes between oxidative stress-related genes and pulmonary fibrosis-related genes. **(D)** Heatmap plot showing the top50 overlapped DEGs between pulmonary fibrosis-related genes and oxidative stress-related genes, highlighting TGF-β1 genes. **(E)** The protein-protein interaction (PPI) network analysis of the “Superoxide anion generation” and “Assembly of collagen fibrils and other multimeric structures” regulated intersection genes performed by STRING and Cytoscape software. **(F)** The mRNA level of α-SMA, COL1α, TGF-β1 expression in the lung tissue of bleomycin-treated mice (n = 12) and vehicle controls (n = 6) determined by real-time fluorescent quantitative PCR. The two indicated groups were compared by Student t-test. *p < 0.05, **p < 0.01, and ***p < 0.001. BLM, bleomycin; PF, pulmonary fibrosis; OS, oxidative stress.

After performing a secondary analysis on a published RNAseq dataset from bleomycin-induced mouse model of pulmonary fibrosis (GSE25640), a total of 3,465 differentially expressed genes (DEGs) was identified in the lung tissue of bleomycin-treated mice compared to controls, with 1764 genes upregulated and 1701 genes downregulated (adjusted p-value cutoff of 0.05) (Figure 1A). Gene set enrichment analysis (GSEA) revealed that “Superoxide anion generation” pathway and “Assembly of collagen fibrils and other multimeric structures” related pathways were among the top 10 upregulated pathways with highest -log10 (p Value) (Figure 1B), supporting the elevated oxidant stress and pulmonary fibrosis characteristics in the lung tissue of bleomycin-induced pulmonary fibrosis. 1,295 genes involved in oxidative stress were obtained from the MitoCarta3.0 database, and 1,589 genes correlated with pulmonary fibrosis were sourced from the Pulmonary Fibrosis Gene Set within the Comparative Toxicogenomics Database. An overlapping set of 500 genes was identified, suggesting an association between oxidative stress and lung fibrosis (Figure 1C). TGF-β1, a pivotal factor in the pathogenesis of fibrosis, was among the top 50 DEGs related to oxidative stress-related lung fibrosis in the GSE25640 dataset (Figure 1D). After functional protein-protein interaction network analysis using String (https://cn.string-db.org/) and Cytoscape, we revealed a TGF-β1-centered interaction network involving the “superoxide anion generation” and “assembly of collagen fibrils and other multimeric structures” pathways within the framework of bleomycin-induced lung fibrosis (Figure 1E). Besides TGF-β1, other markers linked to fibrosis, such as α-SMA and COL1α, also showed increased mRNA levels in the bleomycin-treated cohort (Figure 1F). Together, these results suggest the coexistence of oxidative stress and a TGF-β1-centered pro-fibrotic network in bleomycin-treated mice.

### Bleomycin exposure increases ROS and fibrotic molecules levels in fibroblasts

**FIGURE 2:**
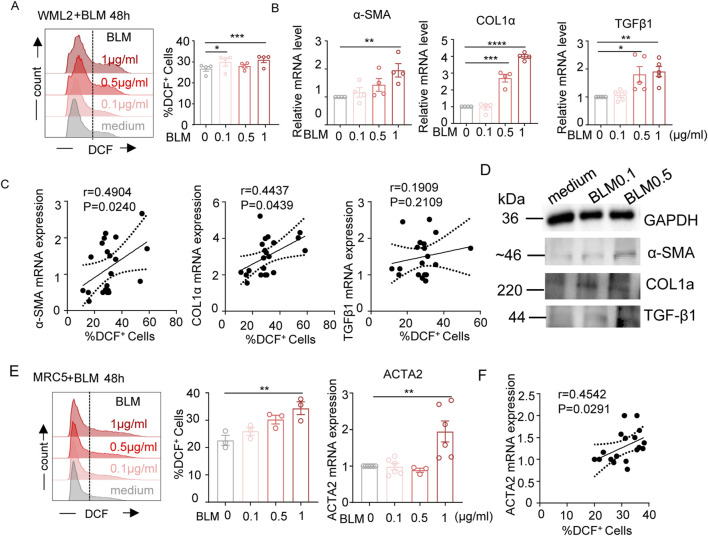
Effects of bleomycin on cellular ROS levels and pro-fibrotic meditators expression in fibroblasts. **(A–D)** WML-2 (mouse fibroblast cells) were exposed to bleomycin with various concentration (0, 0.1, 0.5, and 1 μg/mL) for 48 h. **(A)** Representative histograms and bar graph showing the frequencies of DCF-DA positively stained cells (indicating intracellular ROS levels) (n = 4). **(B)** The relative mRNA levels of fibrosis-related genes measured by real-time fluorescent quantitative PCR (n ≥ 4). **(C)** Spearman correlation between the frequencies of DCF-DA positively stained cells and mRNA levels of α-SMA, COL1α, TGF-β1 in WML-2 cells. **(D)** The protein levels of fibrosis-related molecules including α-SMA, COL1α, and TGF-β1 in indicated groups. **(E)** ACTA2 mRNA levels (left panel) and frequencies of DCF-DA positively stained cells (middle and right panels) in MRC-5 (human fibroblast cells) exposed to various concentration of bleomycin (n ≥ 3/group). **(F)** Spearman correlation between frequencies of DCF-DA positively stained cells and levels of ACTA2 mRNA in MRC-5 cells. The two indicated groups were compared by Student t-test. *p < 0.05, **p < 0.01, and ***p < 0.001.

We next investigated whether bleomycin treatment directly affect fibroblast cells. Mice lung fibroblast WML-2 cells were exposed to bleomycin and evaluated for levels of intracellular ROS and fibrotic markers (Figures 2A–D). The results demonstrated that bleomycin treatment increased intracellular ROS and enhanced the mRNA levels of the fibrosis-related genes α-SMA, COL1α, and TGF-β1 in a concentration-dependent manner (Figures 2A,B). Additionally, the level of intracellular ROS was positively correlated with mRNA levels of α-SMA and COL1α (Figure 2C). Western blot analyses of α-SMA, COL1α, and TGF-β1protein expression further confirmed the upregulation of these pro-fibrotic mediators in the bleomycin-treated mouse lung fibroblast cell line (Figure 2D). Exposure of human fibroblast MRC-5 cells to various concentrations of bleomycin also resulted in upregulation of intracellular ROS level and ACTA2 (encoding α-SMA in human) mRNA level (Figure 2E), showing a positive correlation (Figure 2F). Taken together, these results demonstrate that bleomycin treatment induces oxidative stress and upregulates pro-fibrotic molecules in both mouse and human fibroblast cells.

### Bleomycin exposure raises mitochondrial ROS level and disrupts mitochondrial function in fibroblast with these effects being mitigated by MitoQ

**FIGURE 3:**
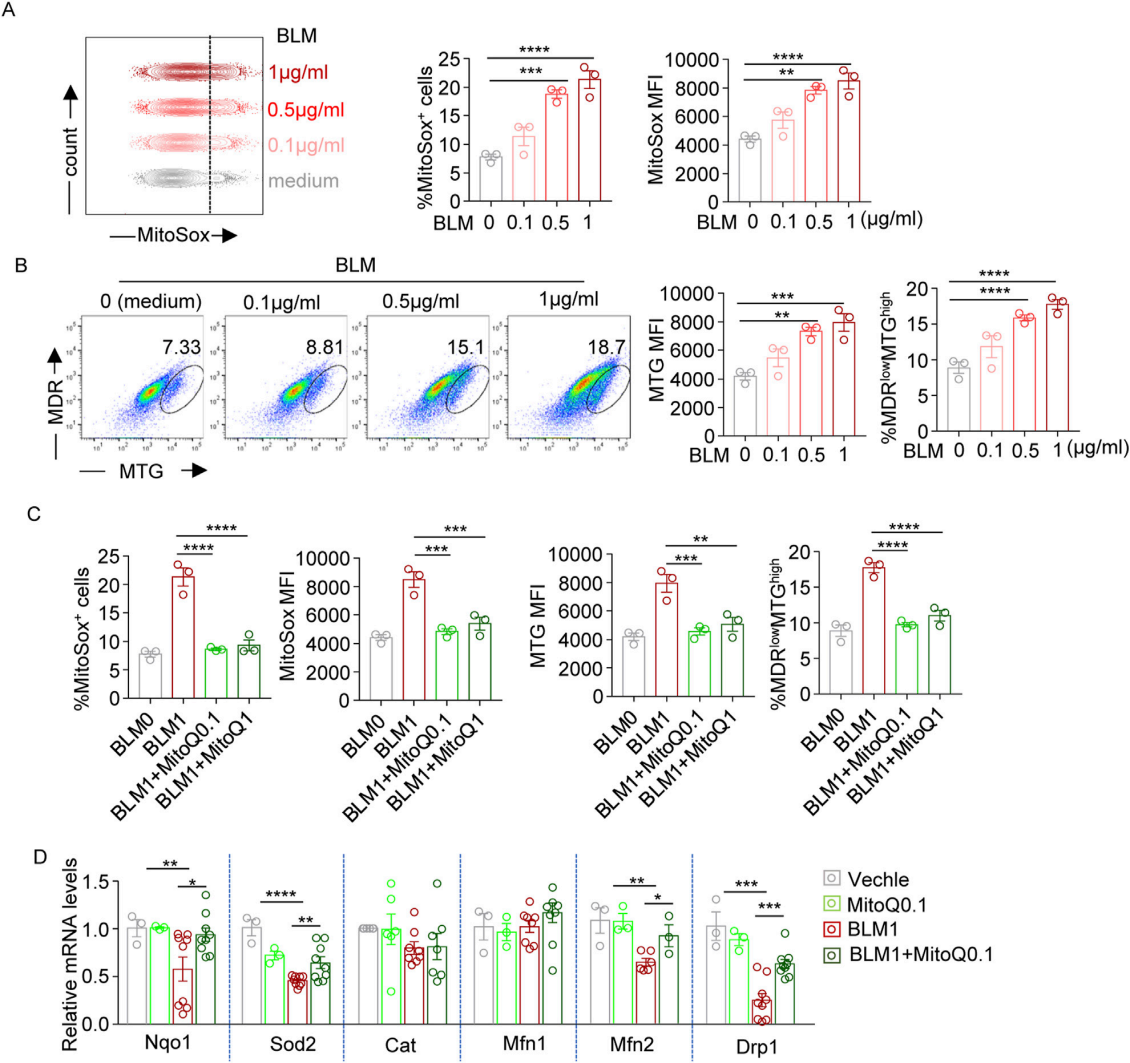
Bleomycin disrupts mitochondrial redox balance and function, while MitoQ restores it. **(A)** Representative histograms and bar graphs showing the frequencies of MitoSox positively stained (MitoSox^+^) cells and mean fluorescence intensity (MFI) of MitoSox staining levels (indicating mitochondrial ROS levels) in WML-2 cells exposed to bleomycin with various concentration (0, 0.1, 0.5, and 1 μg/mL) for 48 h. **(B)** MitoTracker Green (MTG) and MitoTracker Deep Red (MDR) co-staining. Representative dot plot and bar graphs showing MFI of MTG staining levels and the percentages of MDR^low^/MTG^high^ cells in bleomycin-treated WML-2 cells. **(C)** Summary bar graphs showing the frequencies of MitoSox + cells, MitoSox MFI, MRG MFI and the frequencies of MDR^low^/MTG^high^ cells in bleomycin (1 μg/mL, BLM1)-treated WML-2 cells with MitoQ intervention at various concentration (MitoQ0.1:0.1 μM MitoQ; MitoQ1: 1 μM MitoQ). **(D)** The relative mRNA levels of genes involved in ROS scavenging (Nqo1, Sod2, Cat) and mitochondrial function (Mfn1, Mfn2, Drp1) measured by real-time fluorescent quantitative PCR (n ≥ 3) in indicated groups. The two indicated groups were compared by Student t-test. *p < 0.05, **p < 0.01, and ***p < 0.001.

Flow cytometry was performed using MitoSOX Red to detect mitochondrial ROS. Both the percentage of MitoSOX-positive cells and the mean fluorescence intensity (MFI) revealed that bleomycin treatment increased mitochondrial ROS levels in a dose-dependent manner (Figure 3A). Co-staining with MitoTracker Green (MTG, for mitochondrial mass) and MitoTracker Deep Red (MDR, for mitochondrial function) showed bleomycin increased mitochondrial mass and the proportion of MDR^low^MTG^high^ cells in murine lung fibroblasts (Figure 3B), indicating mitochondrial swelling and dysfunction. MitoQ treatment reduced bleomycin-induced mitochondrial ROS generation and attenuated the upregulation of both mitochondrial mass and MDR^low^MTG^high^ cell ratio, suggesting a protective role against these mitochondrial abnormalities (Figure 3C). Furthermore, assessment of mRNA levels for genes involved in ROS scavenging (Nqo1, Sod2, Cat) and mitochondrial function (Mfn1, Mfn2, Drp1) demonstrated that bleomycin reduced the transcript levels of Nqo1, Sod2, Mfn2, and Drp1, which could be recovered by MitoQ intervention (Figure 3D). This indicated that bleomycin altered transcriptional regulation of genes involved in mitochondrial redox balance and function, while MitQ significantly rescued bleomycin-induced mitochondrial dysregulation. Overall, these data collectively illustrate bleomycin’s effects on ROS production and mitochondrial function in fibroblasts, and MitoQ’s beneficial role in mitigating these bleomycin-induced mitochondrial dysfunctions.

### MitoQ is more effective than coenzyme Q in reducing fibrosis-associated molecules that are upregulated by bleomycin or TGFβ1

**FIGURE 4:**
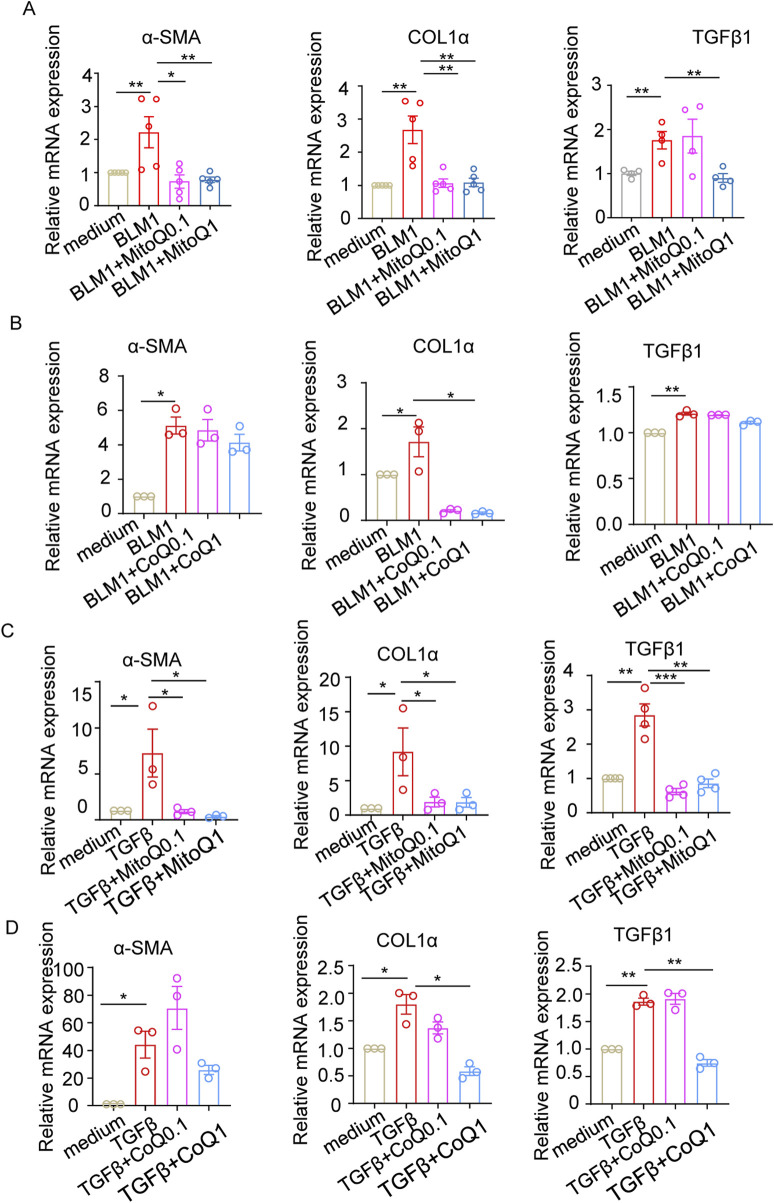
MitoQ and coenzyme Q reduce fibrosis-related molecules upregulated by bleomycin or TGF-β1. WML-2 were exposed to bleomycin (1 μg/mL) or TGF-β1 (5 ng/mL) in the presence of MitoQ or coenzyme Q (0.1 μM or1 μM) (n ≥ 3/group). **(A)** mRNA levels of α-SMA, COL1α, and TGF-β1 in WML-2 cells treated with bleomycin in the presence of MitoQ. **(B)** mRNA levels of α-SMA, COL1α, and TGF-β1 in WML-2 cells treated with bleomycin in the presence of coenzyme Q. **(C)** mRNA levels of α-SMA, COL1α, and TGF-β1 in WML-2 cells treated with TGF-β1 in the presence of MitoQ. **(D)** mRNA levels of α-SMA, COL1α, and TGF-β1 in WML-2 cells treated with TGF-β1 in the presence of coenzyme Q. The two indicated groups were compared by Student t-test. *p < 0.05, **p < 0.01, and ***p < 0.001.

To compare the efficacy of MitoQ and coenzyme Q in mitigating oxidative stress and improving pulmonary fibrosis, WML-2 cells were treated with bleomycin or TGF-β1, while concurrent administrating either MitoQ or coenzyme Q. MitoQ significantly reduced the bleomycin-induced upregulation of α-SMA, COL1α, and TGF-β1 (Figure 4A), while coenzyme Q at an equivalent concentration only inhibited COL1α (Figure 4B). Consistent with the centered role of TGF-β1 in oxidative stress and pro-fibrotic process, TGF-β1 stimulation increased mRNA levels of α-SMA, COL1α, and TGF-β1, which were subsequently diminished by the supplementary of MitoQ (Figure 4C). Although coenzyme Q reduced the mRNA levels of COL1α and TGF-β1 at a relative high concentration, its effect in decreasing α-SMA levels was less prominent (Figure 4D). Together, these findings suggest that mitochondrial-targeted antioxidant MitoQ is more effective than its parent compound, coenzyme Q, at inhibiting the regulation of pro-fibrotic molecules in fibroblasts treated with bleomycin and TGF-β1.

### MitoQ reduced fibrotic index and ROS accumulation in bleomycin-induced pulmonary fibrosis

**FIGURE 5:**
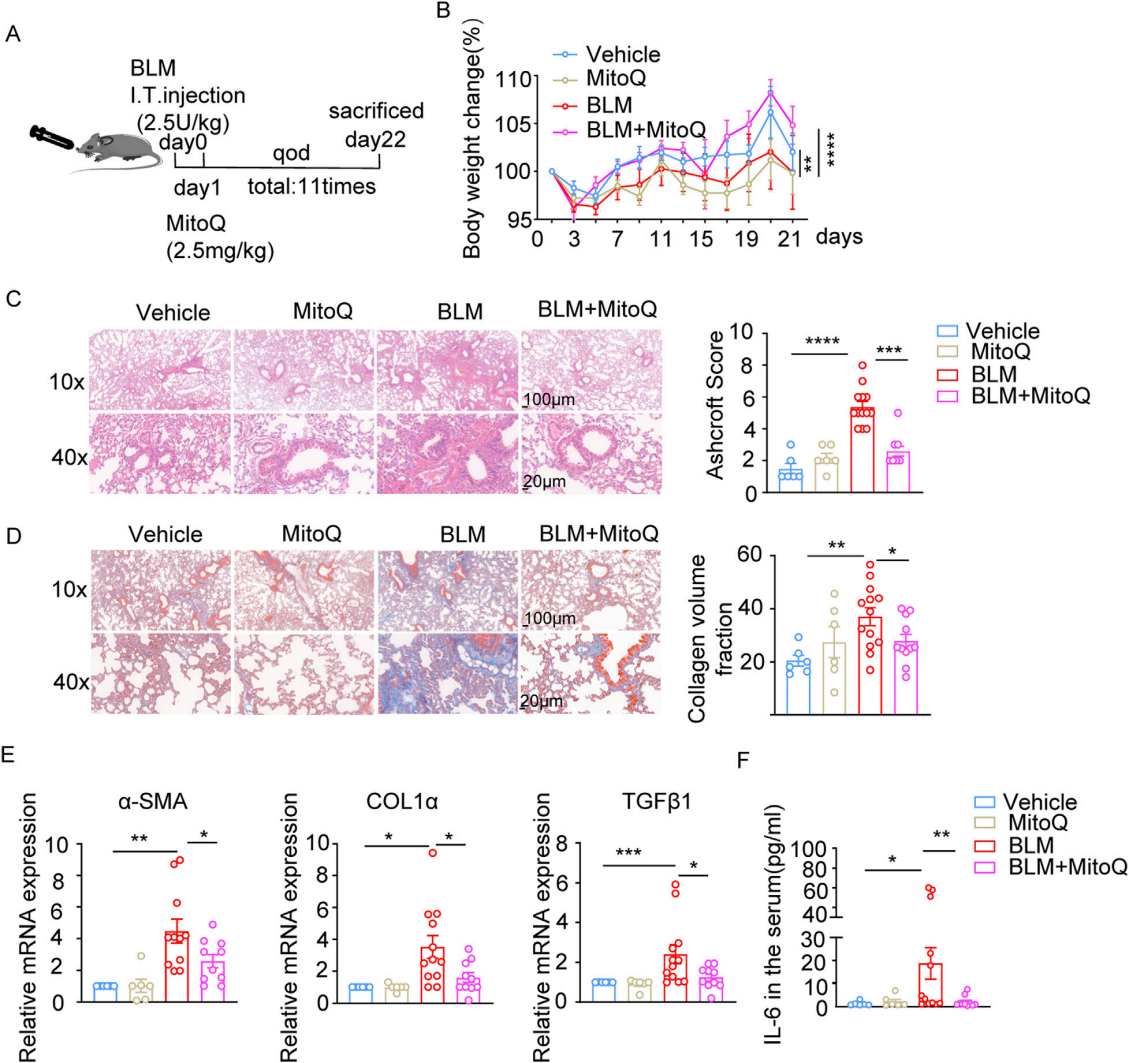
MitoQ reduces fibrotic indices and ROS accumulation in bleomycin-induced lung fibrosis model. **(A)** Experiment schedule. **(B)** Changes in body weight over 21 days (n ≥ 6/group). **(C)** Representative images of HE staining and bar graph showing Ashcroft score in indicated groups; scale bar: 100 µm (top)/20 µm (bottom). **(D)** Representative Masson’s trichrome staining images and bar graph showing collagen volume fraction in indicated groups; scale bar: 100 µm (top)/20 µm (bottom). **(E)** mRNA expression of α-SMA, COL1α, and TGF-β1 in lung tissues determined by real-time PCR in the indicated groups. **(F)** Serum IL-6 cytokines levels in the indicated groups (n ≥ 6). The two indicated groups were compared by Student t-test. *p < 0.05, **p < 0.01, and ***p < 0.001. FI, fluorescence intensity; BLM, bleomycin; ROS, reactive oxygen species.

Next, we assessed the anti-fibrotic and anti-inflammatory properties of MitoQ in the bleomycin-induced pulmonary fibrosis model (Figure 5A). MitoQ administration reversed body weight loss (Figure 5B), reduced the cell infiltration in the alveolar space, alleviated thickening of the alveolar epithelium, and decreased the Ashcroft score in bleomycin-treated mice (Figure 5C). Masson’s trichrome staining showed a significant reduction in collagen deposition in the lung sections from MitoQ-treated mice compared to those without MitoQ treatment in the bleomycin-induced pulmonary fibrosis model (Figure 5D). Notably, MitoQ treatment reduced bleomycin-induced increases in α-SMA, COL1α, and TGF-β1, indicating a decrease in the severity of the pulmonary fibrosis (Figure 5E). In addition, MitoQ treatment lowered the serum levels of proinflammatory cytokine IL-6 (Figure 5F). Taken together, these results suggest that MitoQ, with its mitochondrial-targeting properties, significantly protects the lungs from bleomycin-induced injury and pulmonary fibrosis.

## Discussion

Since oxidative stress plays a key role in the pathogenesis of pulmonary fibrosis, antioxidant therapy presents a promising strategy to scavenge oxygen free radicals and ameliorate deleterious oxidative effects (Otoupalova et al., 2020; Zhu et al., 2021). Although exogenous administration of N-acetylcysteine (NAC), a precursor of glutathione (GSH), has shown efficacy in reducing bleomycin-induced fibrosis in experimental models (Hagiwara et al., 2000; Shahzeidi et al., 1991), its safety and efficacy in humans remain controversial (Calverley et al., 2021; Tomioka et al., 2005). Recent clinical trials have reported that MitoQ, a mitochondria-targeted antioxidant, offers protective benefits in inflammatory diseases without significant side effects (Gane et al., 2010; Snow et al., 2010; Chen K. et al., 2024). Mitochondria are a major source of oxidative stress, and previous work has that mitochondrial-targeted MitoQ ameliorated concanavalin A-induced live damage (Desta et al., 2020). Emerging research has also demonstrated its role in neutralizing oxidative damage in various chronic diseases (Turkseven et al., 2023; Jin et al., 2023; Chen W. et al., 2024; S et al., 2022). Given the promising nature of drugs targeting mitochondrial ROS production, we investigated the efficacy of MitoQ against bleomycin-induced fibrosis and its underlying mechanisms using a mouse model. The data presented here suggest that MitoQ alleviated oxidative stress and reduced inflammation and fibrosis in lung tissues.

Bleomycin, a therapeutic agent with high cure rates in germ cell tumors (Gilligan, 2023; King et al., 2023), is notable for its minimal myelosuppression (Boggs et al., 1974) and immunosuppression effects (Lehane et al., 1975). Understanding how bleomycin causes DNA damage in normal cells may shed light on its enhanced cytotoxicity in specific lung tissues. Several studies have shown that the pulmonary toxicity of bleomycin does not necessarily correlate with its cytotoxic effects on tumor cells (Seidel et al., 2003; Kaminski et al., 2002; Zuo et al., 2002; Kaminski et al., 2000). This is particularly relevant for models induced by bleomycin, even outside of tumor models, to understand the fundamental basis of lung toxicity. To date, little studies have specifically focused on the effect of mitochondrial targeted antioxidant on bleomycin-induced pulmonary fibrosis. To better understand the role of bleomycin-induced ROS accumulation and the resultant cell, protein and DNA damage, we established a bleomycin-induced fibrosis mouse model and treated it with MitoQ to neutralize mitochondrial ROS. Our study demonstrates that treatments with MitoQ reduce collagen accumulation and inflammation in bleomycin-treated mice. While it remains unclear whether mitochondrial dysfunction and oxidative stress are primary events or consequences of inflammation in pulmonary fibrosis development, the beneficial effects of MitoQ seem to correspond to a decrease in oxidative stress. This is evidenced by increased mRNA levels of antioxidant catalase and GPx-1 and protein expression of GPx-1 and Sod1 (19). Consistent with earlier studies, we found that MitoQ not only decreased the level of ROS in fibroblasts but also reduced ROS in whole lung tissues. The decrease of ROS coincided with an alleviation of fibrosis. Taken together, these findings highlight the role of MitoQ in bleomycin-induced pulmonary fibrosis, likely due to its antioxidant properties, reduces inflammation and alleviates pulmonary fibrosis.

TGF-β1, a well-studied pro-fibrotic mediator and a potent driver of myofibroblast differentiation. is regulated by the integrin αvβ6, mainly expressed by epithelia, and its ligand latency-associated peptide (LAP-β1) (Munger et al., 1999; Peng et al., 2022). Once activated, TGF-β1 promotes matrix deposition while dampening the inflammatory response to injury. Two regulatory pathways are involved in this process: TGF-β1 itself induces integrin αvβ6 expression, and TGF-β1 can induce its own expression (Otoupalova et al., 2020). However, in the pathological state of pulmonary fibrosis, persistent TGF-β1 activity and increased ROS disrupt this feedback system, leading to a vicious cycle. Our mouse models demonstrated an increase in TGF-β1 and aggravation of inflammation, as ROS potentiates the expression of TGF-β1 (Liu and Desai, 2015), Sustained activation of TGF-β leads to increased signaling of itself, creating a cycle. The interplay between TGF-β1 and ROS is a major contributor to the maintenance and amplification of the fibrotic process, as observed in systemic sclerosis (Doridot et al., 2019). Studies found that TGF-β1 expression significantly increased specifically in the skin and lungs of mice after bleomycin treatment (Lv et al., 2020; Bai et al., 2020). Our study indicated that both bleomycin and TGF-β1 exposure could induce increased levels of pro-fibrotic molecules, which is thought to be produced primarily by epithelial and macrophage cells (Otoupalova et al., 2020) in lung fibroblast cell lines (Figures 3D, 4C,D). Interestingly, MitoQ not only reduces bleomycin-induced TGF-β1 expression, but also attenuates the TGF-β1-induced upregulation of pro-fibrotic mediators. This suggests that MitoQ’s potential to prevent pulmonary fibrosis by targeting ROS-induced TGF-β1 expression and its downstream effects.

In conclusion, our findings emphasize the elevated mitochondrial ROS levels in bleomycin-induced pulmonary fibrosis, along with the upregulation of Tgfb1-centered oxidative stress-associated fibrosis genes. Mitochondrial-targeted MitoQ demonstrated superior efficacy compared to its parent compound, coenzyme Q, in reducing fibrotic molecule production by fibroblast cells and ameliorating the progression of pulmonary fibrosis.

## Data Availability

The datasets presented in this study can be found in online repositories. The names of the repository/repositories and accession number(s) can be found in the article/supplementary material.

## Abbreviations

BLM, bleomycin; CVF, collagen volume fraction; DEG, differentially expressed gene; ETC., electron transport chain; GSEA, Gene set enrichment analysis; LAP, latency-associated peptide; MitoQ, Mitochondria-targeted ubiquinone; NAC, N-acetylcysteine; NSGCT, non-seminomatous germ cell tumor; qRT-PCR, Quantitative Real-Time Reverse Transcription-Polymerase Chain Reaction; ROS, reactive oxygen species; TPP, triphenylphosphonium; MFI, mean fluorescence intensity.
